# Natural allelic diversities of *GmPrx16* confer drought tolerance in soybean

**DOI:** 10.1111/pbi.14249

**Published:** 2023-11-22

**Authors:** Zhifang Zhang, Junkui Ma, Xia Yang, Zhi Liu, Yucheng Liu, Xueyi Liu, Shan Liang, Zongbiao Duan, Zheng Wang, Xiaoyue Yang, Long Yan, Min Zhang, Shulin Liu, Zhixi Tian

**Affiliations:** ^1^ Key Laboratory of Seed Innovation, Institute of Genetics and Developmental Biology Chinese Academy of Sciences Beijing China; ^2^ The Industrial Crop Institute Shanxi Agriculture University/Shanxi Academy of Agricultural Sciences Taiyuan China; ^3^ The Key Laboratory of Crop Genetics and Breeding, Institute of Cereal and Oil Crops Hebei Academy of Agricultural and Forestry Sciences Shi‐jiazhuang China; ^4^ University of Chinese Academy of Sciences Beijing China; ^5^ Hainan Yazhou Bay Seed Laboratory Sanya China; ^6^ State Key Laboratory of Vegetable Biobreeding, Beijing Vegetable Research Center Beijing Academy of Agriculture and Forestry Science Beijing China

**Keywords:** soybean, drought tolerance, GWAS, peroxidase, natural allelic diversities

Drought has a serious impact on agricultural production, which can result in production losses ranging between 30% and 90%, depending on the crop species (Hussain *et al*., 2019; Lesk *et al*., 2016). Soybean [*Glycine max* (L.) Merr.] is an important crop that provides primary vegetable oil and protein as well as a supplement in livestock feed worldwide (Wilson, 2008). However, soybean is very sensitive to drought (Dong *et al*., 2019), and it has been revealed that from 1983 to 2009 approximately 67 Mha of harvested soybean areas experienced drought stress, resulting in a 7% total yield loss (Kim *et al*., 2019).

To identify the genes and corresponding beneficial alleles conferring drought tolerance in soybean germplasms, we performed genome‐wide association study (GWAS) using a panel of 585 soybean accessions, which had been genotyped in our previous work (Fang *et al*., 2017). The drought tolerance values of these 585 accessions were evaluated in the field in Shanxi Province, China (Figure S1, Table S1). GWAS identified one significant association locus on chromosome 16 ranging from 32 206 964 bp to 32 458 483 bp that covered 23 genes (Figure 1a, Table S2). Haplotype investigation showed that a C to G nonsynonymous SNP in *Glyma.16G164400* (encoding a peroxidase, named as *GmPrx16*) was highly associated with the drought tolerance variation in soybean germplasm, suggesting that *GmPrx16* might be the candidate gene in the association locus on chromosome 16 (Figure 1b). *GmPrx16* was highly expressed in the root and mature leaf tissues (Figure S2a,b). Either dehydration stress treatment or withholding water treatment could induce the transcription of *GmPrx16* (Figure S2c,d). A presence and absence variation (PAV) of a transposable element (TE) existed in the promoter region of *GmPrx16*, which was highly linked to the associated nonsynonymous SNP (Figure S2e). However, we determined that the PAV did not affect the expression of *GmPrx16* in different accessions under normal and drought conditions (Figure S2f,g), indicating that the nonsynonymous SNP instead of the PAV was the causal polymorphism for the functional divergence of *GmPrx16* in soybean.

**Figure 1:**
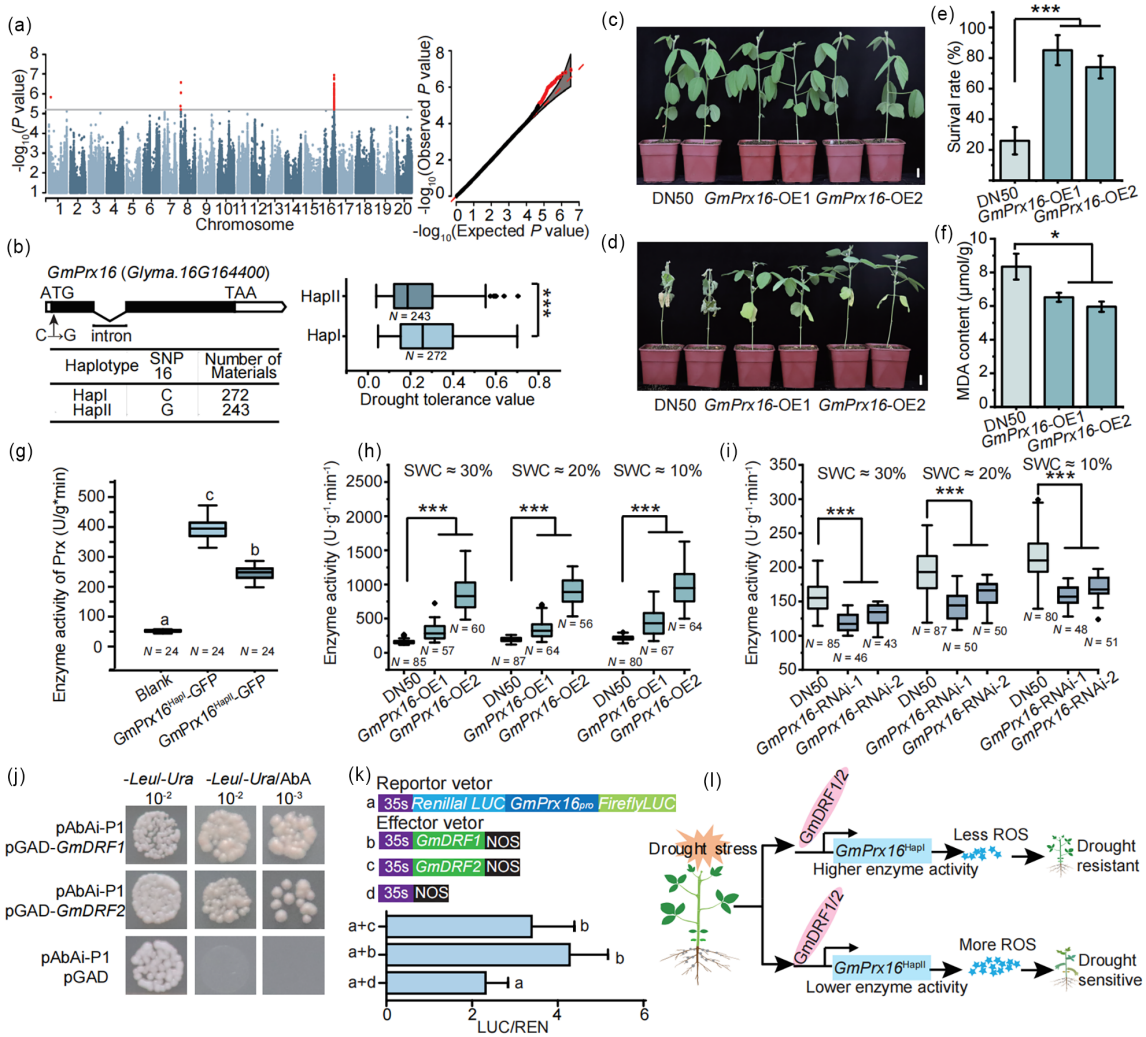
*GmPrx16* confers drought tolerance in soybean. (a) GWAS for drought tolerance value in soybean (significance threshold (−log_10_ (*P* value) = 5.2). (b) Gene structure and haplotype analysis of *GmPrx16*. (c, d) Performance of DN50 and *GmPrx16* overexpression lines (*GmPrx16*‐OE1/2) under well water (c) and drought (d) conditions. Scale bars = 2 cm. (e) Survival rates of DN50 and *GmPrx16*‐OE1/2 after drought treatment. *N* = 9. (f) MDA content in DN50 and *GmPrx16*‐OE1/2 after drought treatment. (g) GmPrx16^HapI^ had higher peroxidase activity than GmPrx16^HapII^. (h, i) Peroxidase activity of *GmPrx16*‐OE1/2 (h) and RNAi lines (i) after drought treatment. SWC indicates soil water content. (j) Y1H assay showing that GmDRF1 and GmDRF2 bind to the promoter of *GmPrx16*. (k) Dual‐LUC assays of the effect of GmDRF1 and GmDRF2 on *GmPrx16* promoter in *Arabidopsis* protoplasts. (l) A proposed working model of *GmPrx16*, GmDRF1 and GmDRF2 in modulating drought tolerance in soybean. *N* indicates the number of samples. Different lowercase letters in (g) and (k) indicate the significance level at 0.05.

To validate the function of *GmPrx16* in conferring drought tolerance in soybean, we performed overexpression (OE) and RNA interference (RNAi) on this gene in Dongnong No. 50 (DN50, an accession harbouring *GmPrx16* ^HapII^) and obtained two independent transgenic lines for each construct (Figure S3a). Under well‐watered condition, no obvious phenotypic differences were observed among DN50, OE lines and RNAi lines (Figure 1c, Figure S3b). When the 2‐week‐old seedling plants were subjected to drought treatment by withholding water for 12‐day, the RNAi lines exhibited obvious drought‐induced wilting compared with DN50 (Figure S3c); after withholding water for 14 days, the OE lines exhibited a higher drought‐tolerant phenotype than DN50 (Figure 1d). Following a 14‐day drought treatment, we rehydrated the plants and found that the survival rates of the OE lines were significantly higher than those of DN50, and the survival rates of the RNAi lines were significantly lower than those of DN50 (Figure 1e, Figure S3d). In consistent, malondialdehyde (MDA) content of DN50 was significantly higher than that of the OE lines, but lower than that of the RNAi lines (Figure 1f, Figure S3e). Interestingly, we also found that the *GmPrx16* OE lines showed improved salt tolerance than DN50 (Figure S3f,g). These results demonstrated that *GmPrx16* may play multiple roles besides drought tolerance in abiotic stress response.

To test the functional divergence of *GmPrx16* ^HapI^ and *GmPrx16* ^HapII^, we expressed the two GmPrx16 haplotypes in tobacco and observed that leaves expressing GmPrx16^HapI^ exhibited higher peroxidase activity than that expressing GmPrx16^HapII^ (Figure 1g). Furthermore, we compared the peroxidase activity of DN50 and the transgenic lines under drought conditions and found that the peroxidase activity in DN50 was significantly lower than that in the OE lines (Figure 1h) and higher than that in the RNAi lines (Figure 1i), consistent with the 3,3′‐diaminobenzidine (DAB) staining assay (Figure S3h,i). To gain a better understanding how *GmPrx16* conferred soybean drought tolerance, we performed RNA sequencing of *GmPrx16* OE and RNAi lines with DN50 under drought conditions (Figure S4a,b). Gene ontology (GO) term enrichment analysis demonstrated *GmPrx16* involved in multiple stress‐responsive pathways (Figure S4c,d). It has been reported that peroxidases involve in lignin biosynthesis (Lee *et al*., 2007). Notably, pathways related to cell wall biosynthesis were also enriched (Figure S4c). We also found that the *GmPrx16* OE lines had higher lignin content and the RNAi lines had lower lignin content than DN50 (Figure S4e). We randomly chose some reported and predicted genes participating in the above pathways and checking their expression levels using qRT‐PCR (Figures S5 and S6). Consistent changing patterns to RNA‐seq were obtained in the transgenic lines, suggesting a reliability of the RNA‐seq data and also indicating that *GmPrx16* influenced multiple pathways under drought stress.

To determine the upstream regulator of *GmPrx16*, we screened a soybean root cDNA library through a one‐hybrid assay (Y1H) and determined that two putative candidate regulators, Glyma.02G085900 (GmDRF1) and Glyma.07G171200 (GmDRF2), could specifically bind the promoter of *GmPrx16* (Figure 1j). Transcriptional analysis showed that *GmDRF1*/*2* were mainly expressed in leaves (Figure S7a) and either dehydration stress treatment or withholding water treatment could induce the transcription of *GmDRF1*/*2* (Figure S7b,c). Transient transfection assays in *Arabidopsis* protoplasts and tobacco leaves indicated that GmDRF1/2 could significantly induce the expression of *GmPrx16* (Figure 1k, Figure S7d). Therefore, we propose a model to illustrate the molecular mechanisms of *GmPrx16* in modulating soybean drought tolerance (Figure 1l): GmDRF1/2 physically bind to the promoter of *GmPrx16* and promote its expression, and drought stress induces the expression of *GmDRF1*/*2*, thereby influencing the accumulation of ROS and multiple stress response pathways.

Taken together, we identified *GmPrx16* as a key gene responsible for drought tolerance in soybean natural populations, providing insights into the development of drought‐tolerant soybean cultivars by molecular design breeding.

## Conflict of interest

The authors declare no conflicts of interest.

## Author contributions

Z.T. designed the research; Z.Z., J.M., X.L., S.L., X.Y., Y.L., Z.L., S.L., Z.D., Z.W., X.Y., L.Y., and M.Z. performed the research; Z.Z., S.L., and Z.T. analysed the data; Z.Z., S.L., and Z.T. wrote the manuscript. All authors reviewed the manuscript.

## Supporting information


**Figure S1** Frequency distribution histogram of drought tolerance value.
**Figure S2** *GmPrx16* is the causal gene on the chromosome 16 association locus for drought tolerance in soybean.
**Figure S3** *GmPrx16* confers drought and salt tolerance through regulating peroxidase activity in soybean.
**Figure S4** *GmPrx16* affects multiple signaling pathways under drought condition in soybean.
**Figure S5** Expression levels of reported genes participating in drought tolerance in soybean in *GmPrx16* transgenic lines and DN50 under drought condition.
**Figure S6** Expression levels of representative genes participating in the hormone signaling pathway and cell wall biosynthesis in DN50 and *GmPrx16* transgenic lines under drought condition.
**Figure S7** GmDRF1 and GmDRF2 regulate *GmPrx16* transcription.

**Table S1** Information for the natural population used in the GWAS.
**Table S2** Candidate genes list in the locus located on chomosome 16.
