# Supplementary material for: Natural allelic diversities of GmPrx16 confer drought tolerance in soybean
Source: Plant Biotechnol J. 2023 Nov 22;22(3):535–7. doi: 10.1111/pbi.14249 (PMC10893949; doi:10.1111/pbi.14249)
Supplement: Supplementary file 1 — Figure S1 Frequency distribution histogram of drought tolerance value. Figure S2 GmPrx16 is the causal gene on the chromosome 16 association locus for drought tolerance in soybean. Figure S3 GmPrx16 confers drought and salt tolerance through regulating peroxidase activity in soybean. Figure S4 GmPrx16 affects multiple signaling pathways under drought condition in soybean. Figure S5 Expression levels of reported genes participating in drought tolerance in soybean in GmPrx16 transgenic lines and DN50 under drought condition. Figure S6 Expression levels of representative genes participating in the hormone signaling pathway and cell wall biosynthesis in DN50 and GmPrx16 transgenic lines under drought condition. Figure S7 GmDRF1 and GmDRF2 regulate GmPrx16 transcription. [file PBI-22-535-s002.docx]

**
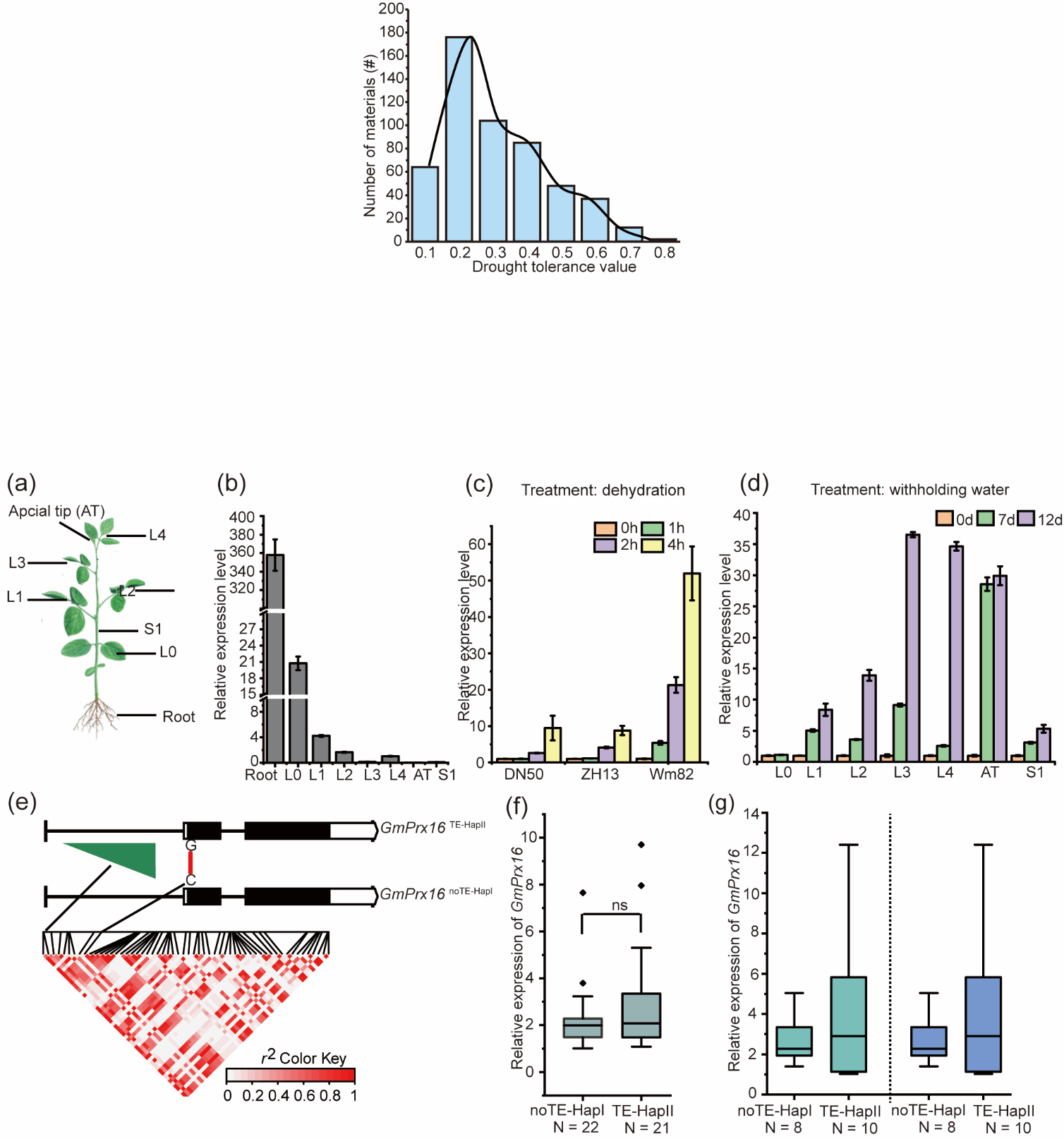
**

**Figure S1** Frequency distribution histogram of drought tolerance value.

**
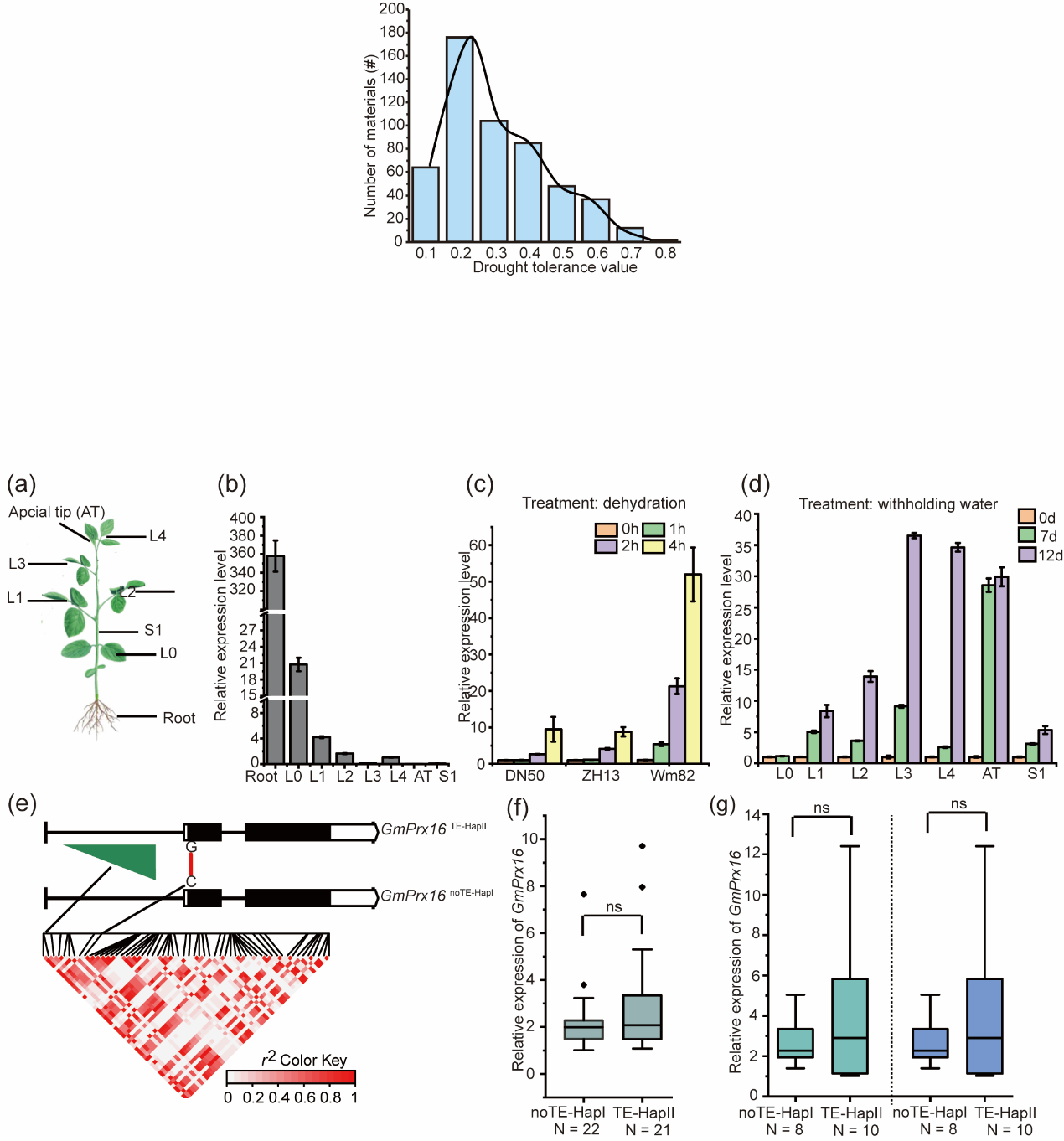
**

**Figure S2** *GmPrx16* is the causal gene on the chromosome 16 association locus for drought tolerance in soybean. (a) Tissues from different parts of soybean DN50 for tissue-specific expression analysis. (b) Expression pattern of *GmPrx16* in different plant tissues in DN50. (c) *GmPrx16* expression is induced by dehydration stress in three accessions, DN50, Zhonghuang No.13 (ZH13) and Williams 82 (Wm82). 0/1/2/4 h indicate the hours for treatment. (d) Expression pattern of *GmPrx16* in different plant tissues in DN50 after withholding-water treatment for 0 day (0d), 7 days (7d) and 12 days (12d). (e) Gene structure (promoter and CDS) of *GmPrx16* (top) and LD heatmap (bottom). The green triangle indicates the TE in the promoter, black frame indicates the exon in the CDS, *r^2^* indicates the Pearson coefficient of correlation. TE insertion (TE) and deletion (noTE) with SNP variation in the first exon (HapI/II) combined two new haplotypes (TE-HapII and noTE-HapI). (f) Expression level of *GmPrx16* in soybean accessions with different haplotypes. (g) Expression level of *GmPrx16* in soybean accessions with different haplotypes under dehydration stress treatment for 2 h (left) and 4 h (right). N indicates the number of accessions. ns means non-significant differences.


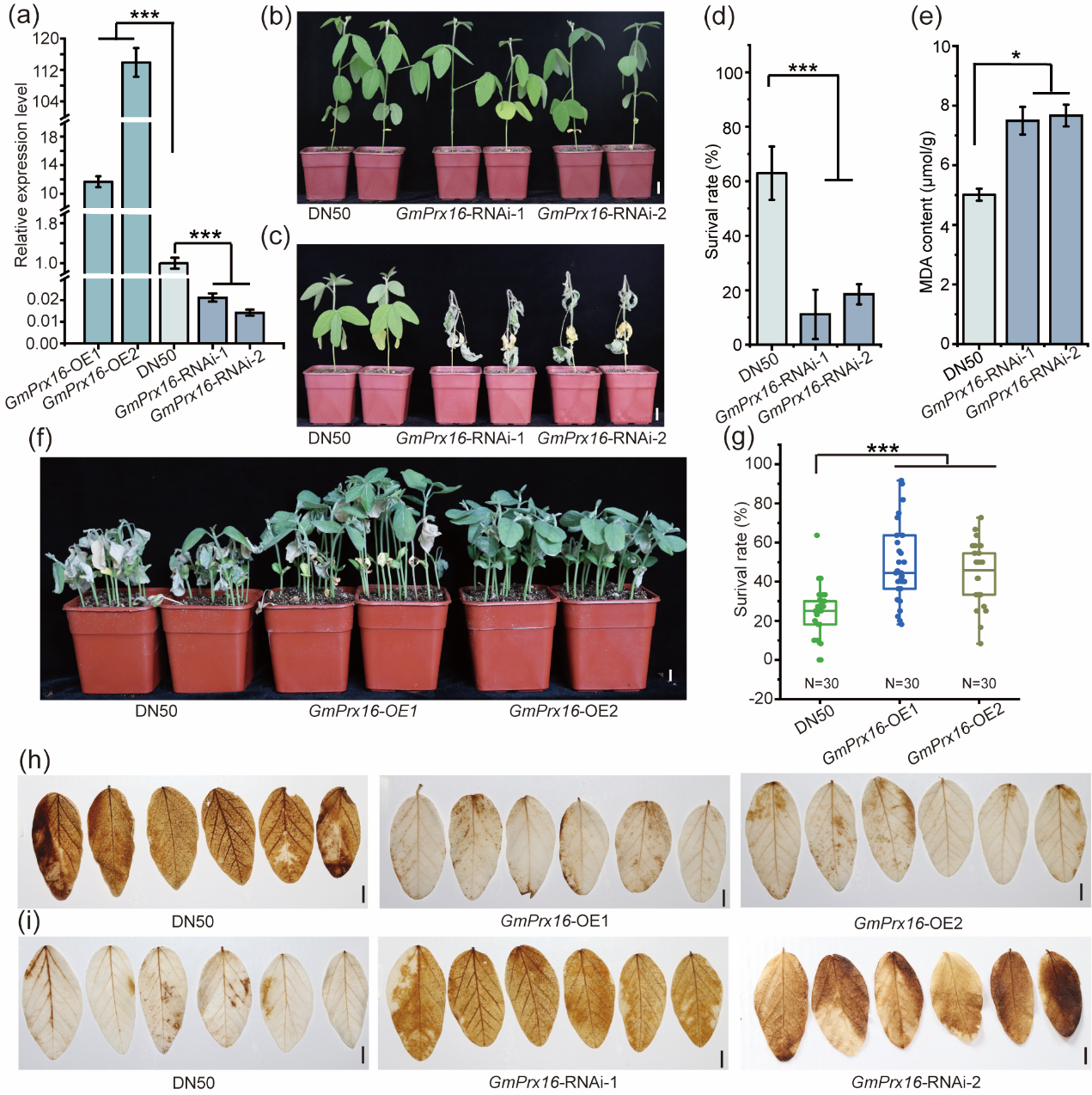


**Figure S3** *GmPrx16* confers drought and salt tolerance through regulating peroxidase activity in soybean. (a) Expression of *GmPrx16* in DN50 and transgenic lines. (b) Performance of DN50 and *GmPrx16* RNAi lines (*GmPrx16*-RNAi-1/2) under well water condition. Scale bars = 2 cm. (c) Performance of *GmPrx16* RNAi lines under drought conditions. Scale bars = 2 cm. (d) Survival rates of *GmPrx16* RNAi lines after drought treatment. N = 9. (e) MDA content in *GmPrx16* RNAi lines after drought treatment. (f) *GmPrx16* overexpression lines show salt tolerance in soybean, scale bars = 1 cm. (g) Survival rate of *GmPrx16* overexpression lines under salt stress in soybean. N indicates the number of samples. (h), (i) DAB staining reveals the H_2_O_2_ content of the leaves of *GmPrx16* overexpression lines (h) and RNAi lines (i) after drought treatment, scale bar = 1 cm. *, *P* value < 0.05, ***, *P* value < 0.001.


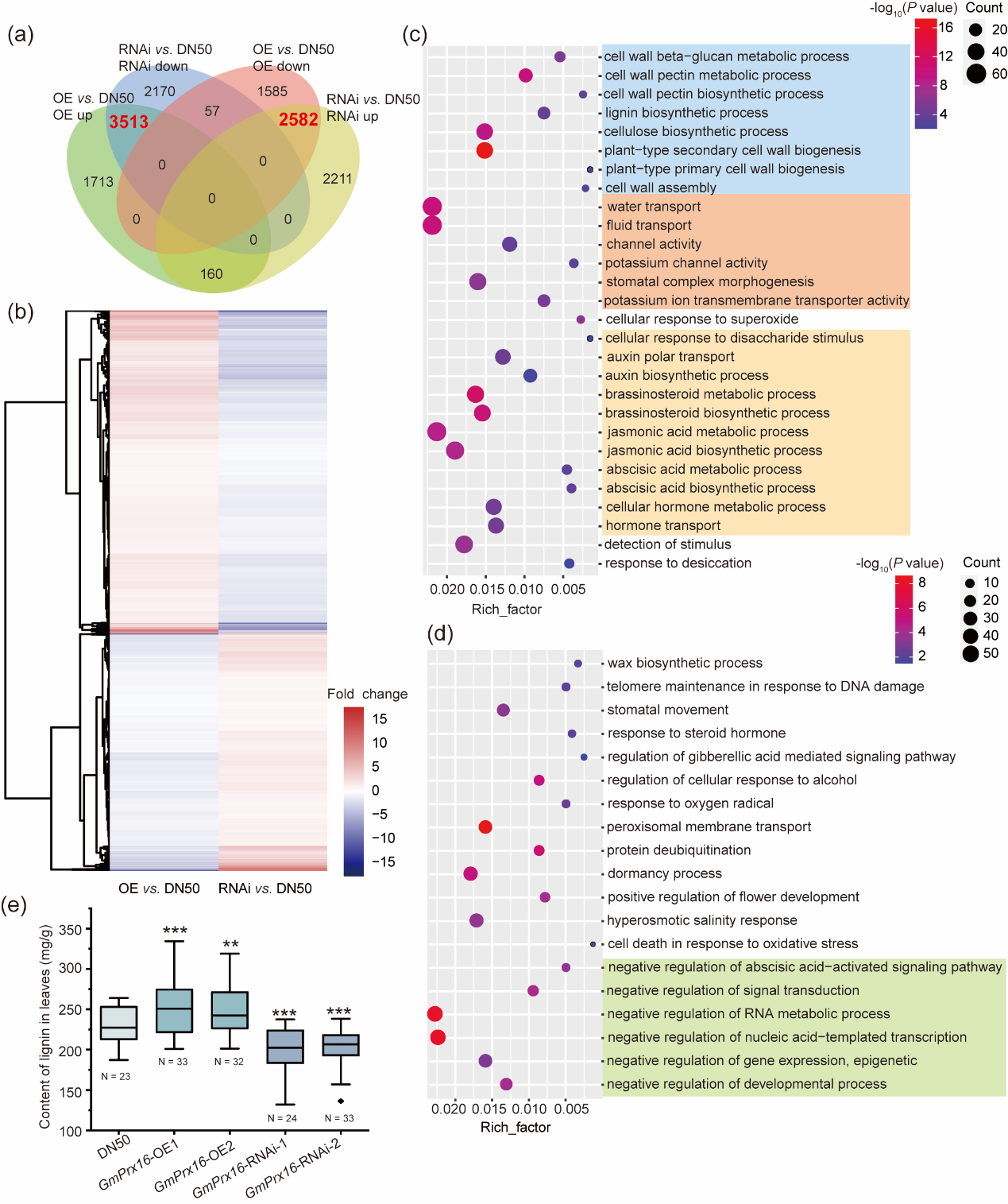


**Figure S4** *GmPrx16* affects multiple signaling pathways under drought condition in soybean. (a) Differentially expressed genes (DEGs) among DN50 and *GmPrx16* transgenic lines. (b) Heatmap of DEGs among DN50 and *GmPrx16* transgenic lines under drought condition. (c) Gene ontology term enrichment of the DEGs that up-regulated in *GmPrx16* OE lines and down-regulated in *GmPrx16* RNAi lines. (d) Gene ontology term enrichment of the DEGs that down-regulated in *GmPrx16* OE lines and up-regulated in *GmPrx16* RNAi lines. (e) Lignin content in DN50 and *GmPrx16* transgenic lines. **, *P* value < 0.01, ***, *P* value < 0.001, N means the number of samples.


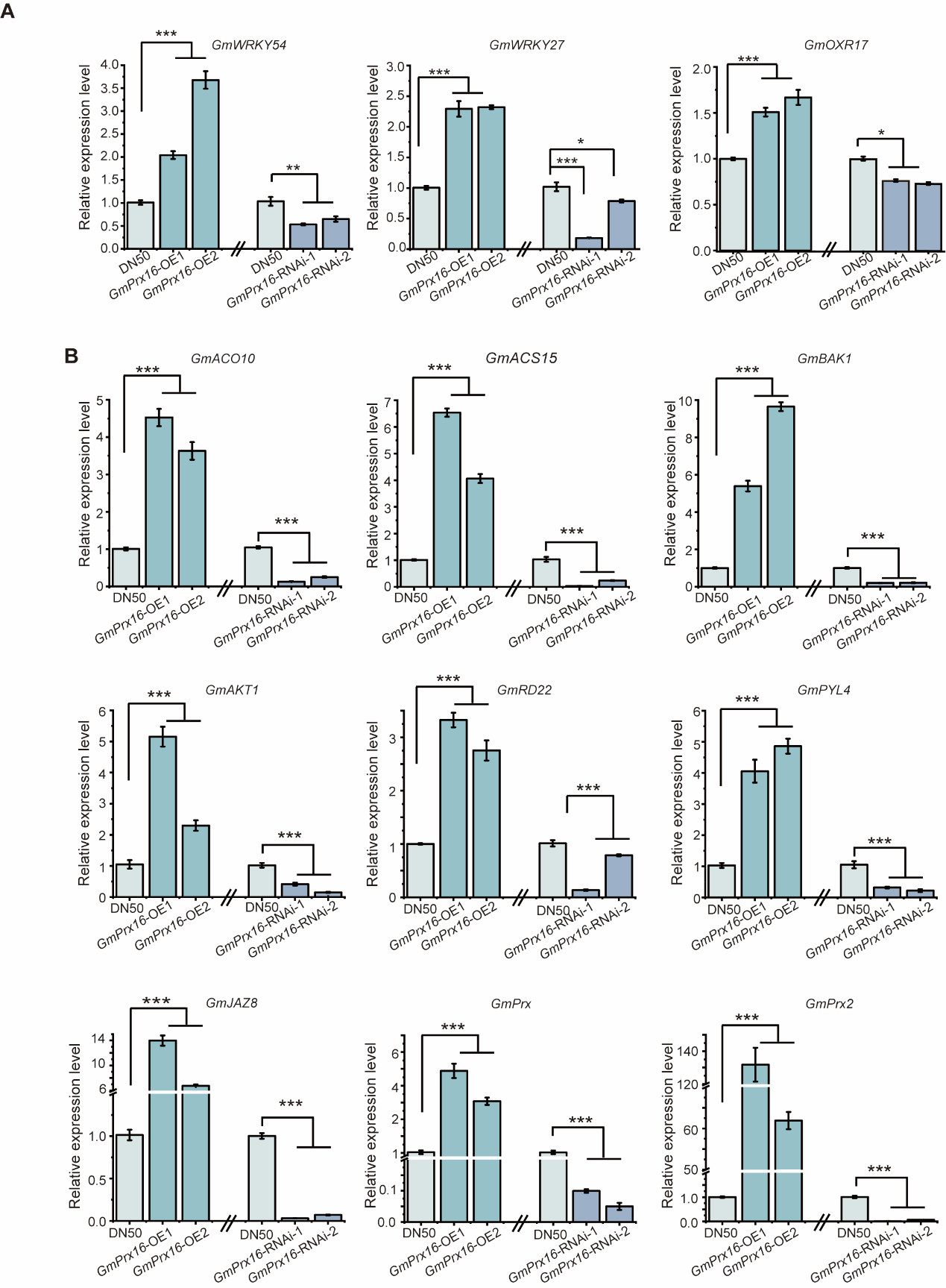


**Figure S5** Expression levels of reported genes participating in drought tolerance in soybean in *GmPrx16* transgenic lines and DN50 under drought condition. *, *P* value < 0.05; ***, *P* value < 0.001.


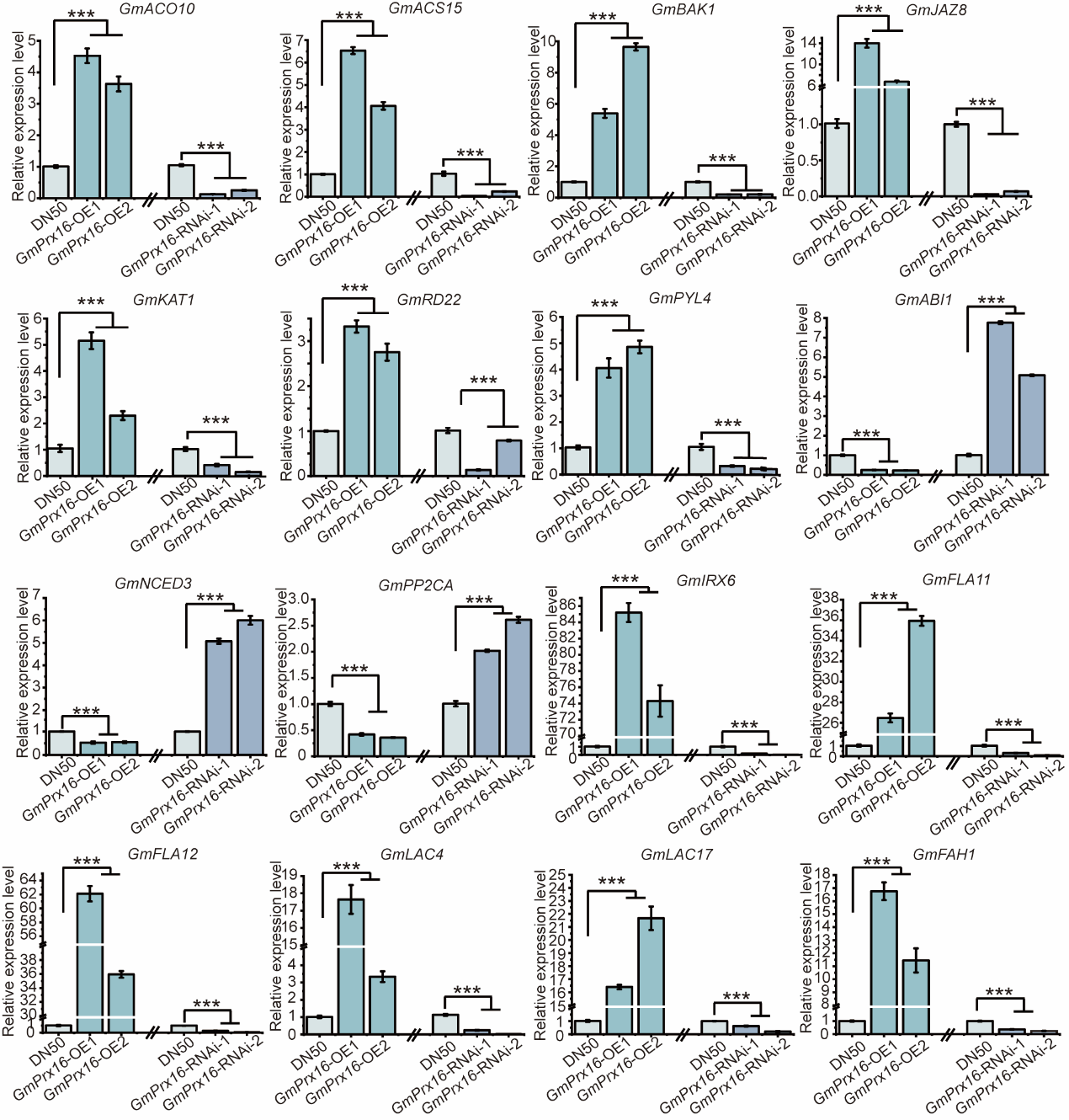


**Figure S6** Expression levels of representative genes participating in the hormone signaling pathway and cell wall biosynthesis in DN50 and *GmPrx16* transgenic lines under drought condition. *GmACO10* and *GmACS15* encode key enzymes in ethylene biosynthesis; *GmBAK1* participates in brassinolide signaling; *GmJAZ8* participates in jasmonic acid signaling; *GmRD22*, *GmPYL4*, *GmABI1*, *GmNCED3*, and *GmPP2CA* participate in the ABA biosynthesis and signaling pathway. *GmIRX6* involves in secondary cell wall biosynthesis; *GmFLA11* and *GmFLA12* participate in cellulose biosynthesis; *GmLAC4*, *GmLAC17* and *GmFAH1* participate in lignin biosynthesis. ***, *P* value < 0.001.


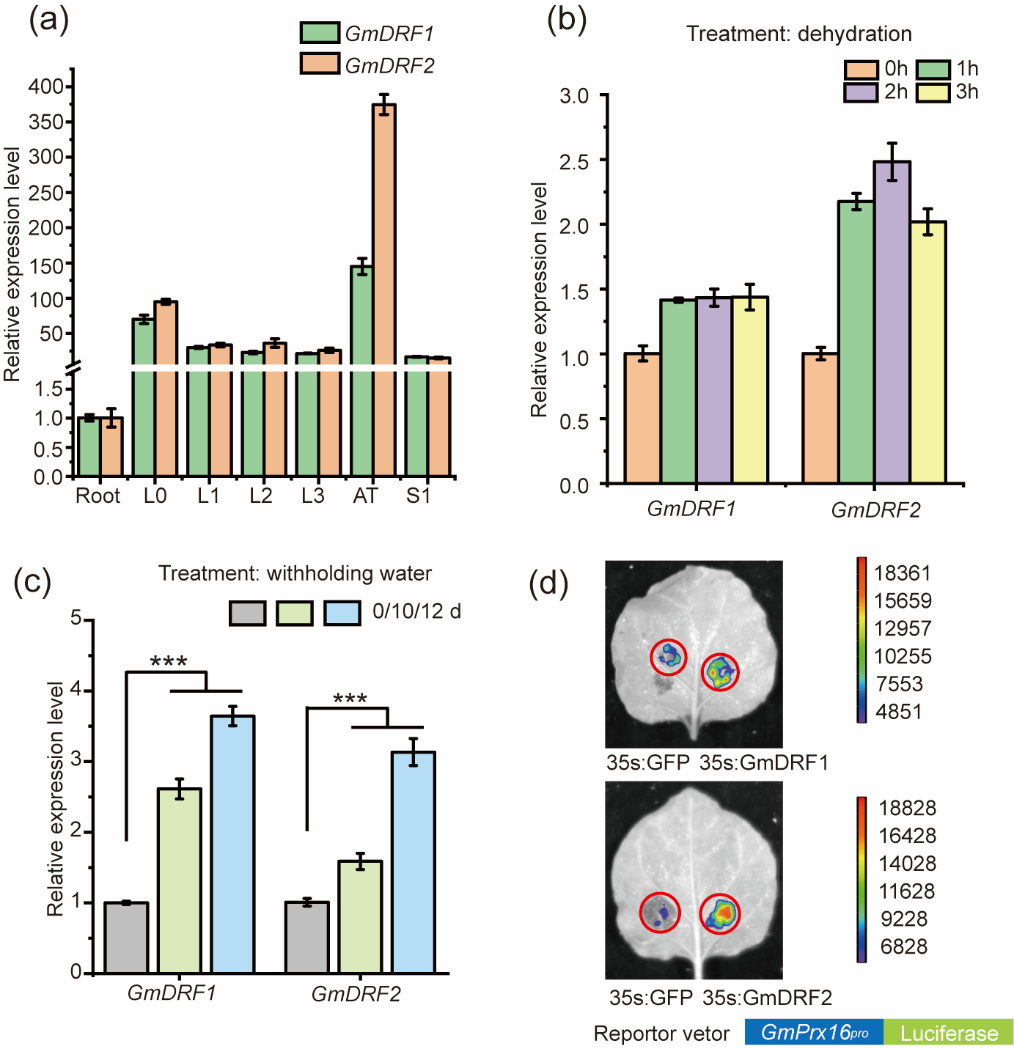


**Figure S7** GmDRF1 and GmDRF2 regulate *GmPrx16* transcription. (a) Tissue-specific expression of *GmDRF1*/*2* in DN50. (b) *GmDRF1*/*2* expression were induced by dehydration stress in Wm82, 0/1/2/3 h indicate the hours for treatment. (c) *GmDRF1*/*2* expression levels are up-regulated by drought treatment. ***, *P* value < 0.001. (d) Luciferase imaging assays in *Nicotiana benthamiana.* *Agrobacterium* carrying different plasmids are co-expressed with the reporter. The colors represent the interaction strength.
